# Effects of over-expression of TLR2 in transgenic goats on pathogen clearance and role of up-regulation of lysozyme secretion and infiltration of inflammatory cells

**DOI:** 10.1186/1746-6148-8-196

**Published:** 2012-10-22

**Authors:** Shoulong Deng, Kun Yu, Baolu Zhang, Yuchang Yao, Yufeng Liu, Haijuan He, Hongtao Zhang, Maosheng Cui, Juncai Fu, Zhengxing Lian, Ning Li

**Affiliations:** 1 National key Lab of Agro-biotechnology, China Agricultural University, Beijing, 100193, People’s Republic of China; 2 Laboratory of Animal Genetics and Breeding, College of Animal Science and Technology, China Agricultural University, Beijing, 100193, People’s Republic of China; 3 National key Lab of Agro-biotechnology, Beijing, 100193, People’s Republic of China; 4 College of Animal Science and Technology, Northeast Agricultural University, Harbin, 150030, People’s Republic of China; 5 Animal Husbandry Research Institute of HeilongjiangAcademy of Agricultural Sciences, Harbin, 150086, People’s Republic of China; 6 College of Life Science and Technology, HeilongjiangBa Yi Agricultural University, Daqing, 163000, People’s Republic of China; 7 Tianjin Institute of Animal Sciences, Tianjin, 300112, People’s Republic of China

**Keywords:** Goats, TLR2, Over-expression, Pam3CSK4

## Abstract

**Background:**

Toll-like receptor 2 (TLR2) is important to host recognition of invading gram-positive microbes. In goats, these microbes can cause serious mastitis, anthrax, tetanus, and other problems. Transgenic goats constitutively over-expressing TLR2 in many tissues serve as a suitable model for the study of the role of TLR2 over-expression in bacterial clearance.

**Results:**

*Capra hircus* TLR2 over-expression vector (p3S-LoxP-TLR2) was used to generate transgenic goats by egg microinjection. The integration efficiency was 8.57%. Real-time PCR and immunohistochemical results confirmed that the goats over-expressing the TLR2 gene (Tg) expressed more TLR2 than wild-type goats (WT). Monocyte-macrophages from the bloodstreams of transgenic goats were stimulated with synthetic bacterial lipoprotein (Pam3CSK4) and by the promotion of interleukin-6 (IL-6) and IL-10 expression in vitro. The oxidative damage was significantly reduced, and lysozyme (LZM) secretion was found to be up-regulated. Ear tissue samples from transgenic goats that had been stimulated with Pam3CSK4 via hypodermic injection showed that transgenic individuals can undergo the inflammation response very quickly.

**Conclusions:**

Over-expression of TLR2 was found to decrease radical damage to host cells through low-level production of NO and MDA and to promote the clearance of invasive bacteria by up-regulating lysozyme secretion and filtration of inflammatory cells to the infected site.

## Background

Most gram-positive bacteria, such as *Bacillus anthracis*, *M. tuberculosis*, *Staphylococcus aureus*, and *Clostridiumtetani*, are harmful. They cause a wide range of diseases in both immunocompetent and immunocompromized hosts. Many of them can cause serious health problems in goats. These include mastitis, erysipelas, listeriosis, anthrax, encephalitis, and spontaneous abortion. Toll-like receptors (TLRs) are type I trans-membrane proteins. They are ligands of a large number of endogenous and exogenous targets. TLR2 is considered the main pattern receptor of various gram-positive bacteria—including mycobacteria, viruses, and their products [1]. TLR2 can form heterodimers with both TLR1 and TLR6. These heterodimers play a key role in innate immunity by binding pathogen-associated molecular patterns (PAMP) and causing an inflammatory cascade reaction [2]. This activates monocytes and macrophages and promotes neutrophil infiltration [3]. The signalling pathway initiated by TLR2 is called the myeloid-differentiation-factor88-dependent pathway (MyD88-dependent pathway) [4,5]. TLR2 is secreted in a wide variety of tissues and organs, but not all of them show the same levels of expression [6]. Under challenge, TLR2 expression is up-regulated. This results in translocation of nuclear factor (NF-κB), which in turn causes the production of pro-inflammatory cytokines, such as interleukin-6 (IL-6) and nitric oxide (NO). Studies have shown that TLR2 acts as an agonist by modulating the immune response and Th1/Th2 balance [7]. IL-6 is a pro-inflammatory cytokine. It is produced by Th2 cells and acts as a mediator of acute phase response. It accelerates the infiltration of inflammatory cells. Cytokines such as IFN-γ, which is produced by Th1 cells, inhibit the differentiation of Th2 cells. IL-10, an immune regulation cytokine produced by monocytes and macrophages, is crucial to Th1/Th2 balance [8]. Several types of immune dysfunction can occur if this balance is disrupted. During pathogen infection, a healthy Th1/Th2 balance can facilitate an effective host response. Lysozyme (LZM) is especially active against gram-positive bacteria. Lysozyme breaks down the carbohydrate chains on their surfaces, destroying the structural integrity of the cell wall. The bacteria then burst under their own internal pressure.

Synthetic bacterial lipopeptide Pam3CSK4 is a TLR2 agonist. It is a heterodimer of TLR1 and TLR2 [9]. TLR2 serves as the recognizing receptor for gram-positive bacteria. Study showed TLR2 expression up-regulated under Pam3CSK4 challenge.

To determine the effect of over-expression of TLR2 on pathogen resistance, TLR2 transgenic goats were generated. Pam3CSK4 was used as a TLR2 activator. NO, MDA, and LZM products were detected and used to evaluate the immune response. Further experiments were performed out to determine the effects of over-expression of TLR2 on the immune systems of goats both in vivo and in vitro. Tissue sections were evaluated histological. The blood biochemical parameters of the experimental goats were characterized.

## Results

### Production of transgenic goats over-expressing *Capra hircus*TLR2

**Figure 1:**
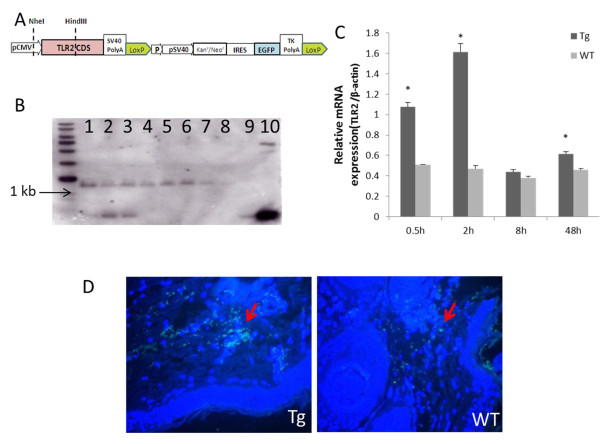
**Transgenic goats over**-**expressing *Capra hircus *TLR2.** (**A**) Structure of p3S-LoxP-TLR2 plasmids.** (B**) Southern blotting analysis, 1–3: positive samples; 4–7: negative samples; 8: control; 9, 10: p3S-Loxp-TLR2 plasmid. (**C**) Relative quantitative analysis of TLR2 mRNA expression under Pam3CSK4 stimulation. (**D**) Immunohistochemical study of TLR2 protein product: transgenic (Tg) group, wild-type (WT) group. Sections were double-stained with DAPI (blue) and TLR2-FITC (green).

*Capra hircus* TLR2 cDNA was generated using RT-PCR. A 2365 bp fragment was amplified. This fragment was linked to double-enzyme-digested p3S-LoxP vector. Two LoxP sequences were introduced. An expression vector, p3S-LoxP-TLR2, was constructed (Figure 1A). As shown in Table 1, 5 pL of linearized plasmid was microinjected into 221 embryos in total, and these injected embryos were immediately transferred into 40 recipients, producing 35 goats. Southern blot analysis confirmed that 3 of these goats were transgenic. The dose of 5 ng/μL was found to be more efficient than 10 ng/μL (Table 1 and Figure 1B). Real-time PCR analysis was performed to determine levels of TLR2 transcription. During the experiment, TLR2 expression in Tg goats was higher than in control goats (Figure 1C). The amount of TLR2 mRNA in transgenic goats (Tg) was significantly higher than in wild-type goats (WT) at 0.5, 2, and 48 hours (*P<0.05*). In Tg, TLR2 transcription reached its peak 2 hours after challenge. Immunohistochemical results confirmed that the Tg group expressed more TLR2 than the WT group (Figure 1D).

**Table 1:** **Microinjection production of over**-**expression of TLR2 gene in goats**

**Gene concentration**	**No**. **of donors**	**No**. **of micro**-**injections**	**No**. **of ET recipients**	**No**. **of goats**	**Southern**-**positive rate(%)**
5 ng/μL	8	99	19	10	10.00 (1/10)
10 ng/μL	10	122	21	25	8.00 (2/25)
Total	18	221	40	35	8.57 (3/35)

Note: No. =number.

### Normal blood biochemical parameters of transgenic goats

There was no statistically significant difference between transgenic goats and non-transgenic goats with respect to routine blood and serum biochemical parameters at 120 days (Tables 2 and 3).

**Table 2:** **Routine blood examinations of wild**-**type and TLR2 transgenic goats**

**Blood routine**	**Tg**	**WT**
WBC (10^9^/L)	14.59±2.7	19.91±7.33
RBC (10^12^/L)	4.13±0.52	4.01±0.26
HGB (g/L)	102.67±7.0	108±3.61
HCT (%)	13.83±1.72	13.57±0.85

**Table 3:** **Blood biochemistry of wild**-**type and TLR2 transgenic goats**

**Blood biochemistry**	**Tg**	**WT**
TP (g/L)	69.07±11.45	73.27±12.78
Alb (g/L)	24.1±2.55	25.3±0.95
Glo (g/L)	45.3±9.29	47.97±12.92
ALT (μ/L)	39±15.39	45.33±6.03
AST (μ/L)	88.33±21.78	94.33±9.5
CRP (mg/L)	1.81±0.42	1.72±0.31
BUN (mmol/L)	6.67±2.16	7.88±0.44
Glu (mmol/L)	4.51±0.18	3.77±0.65
TG (mmol/L)	0.38±0.19	0.42±0.16

### Promotion of IL-6 and IL-10 expression in transgenic goats

**Figure 2:**
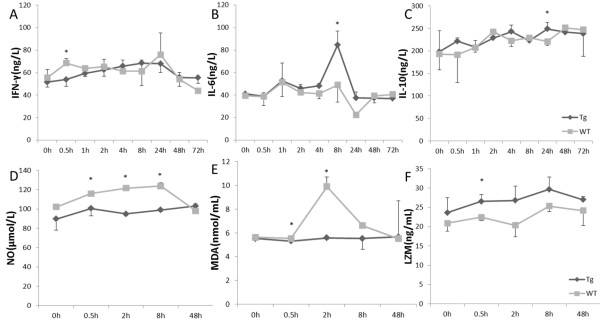
**Transgenic goats over**-**expressing *Capra hircus *TLR2 monocytes and macrophages under Pam3CSK4 stimulation.** Promotion of IL-6 and IL-10 expression in transgenic goats, ELISA testing of changes in inflammatory cytokines: (**A**) IFN-γ, (**B**) IL-6, and (**C**) IL-10 expression.(**D**, **E**) Prevention oxidative damage in transgenic goats, levels of NO and MDA expression. (**F**) Up-regulation of lysozyme secretion in transgenic goats by ELISA. Data are means ± SE. * Values within the same concentration of Pam3CSK4 (1 μg/mL) were found to differ significantly between different groups at different times (*P<0.05*). Tg = transgenic group, WT = wild-type group.

Under Pam3CSK4 stimulation, levels of secreted IFN-γ were significantly lower in the Tg group than in the WT group at 0.5 hours. During the experiment, IFN-γ expression was unregulated in Tg, and this pattern remained relatively stable. Peak IFN-γ expression was observed at 8 hours post stimulation in Tg, which is earlier than in the WT group. The expression of IFN-γ in the WT group showed a fluctuating uptrend, including two points of up-regulation. The peak concentration of the WT group was observed 24 hours after stimulation. It was also higher than the peak concentration observed in the Tg group. At 8 hours, IL-6 levels were higher in the Tg group than in the WT group (*P<0.05*). This may have caused an acute inflammatory response. In the Tg group, IL-10 expression reached its highest level at 24 hours and then became down-regulated (Figure 2A, B, and C).

In the WT group, IFN-γ levels increased during the first 0.5 hours, but IL-10 expression was up-regulated immediately. High levels of IFN-γ expression were observed 24 hours after challenge. In contrast, IL-10 and IL-6 expression decreased, and minimal levels of IL-6 were observed. Soon after that, IL-10 expression increased again. This indicates that the inflammatory response was being suppressed.

In the Tg group, there was an inflammatory imbalance between IL-10 and IL-6 when IL-6 and IFN-γ levels peaked at 8 hours and subsequently decreased. This suggests that a strong inflammatory reaction occurred. The highest IL-10 level was detected at 24 hours. This indicates that the inflammatory reaction was inhibited. These results suggest that over-expression of TLR2 might regulate the expression of Th2 cytokines and trigger the inflammatory response.

### Prevention of oxidative damage in transgenic goats

In the immune response, NO acts as a functional cellular signalling molecule. In macrophages, nitric oxide synthase (NOS) is positively regulated by IFN-γ. MDA is one of the most frequently used indicators of lipid per-oxidation. In this study, the concentrations of NO and MDA in the WT group were higher than in the Tg group at 0.5 hours and 2 hours (*P*<0.05), and they remained relatively low and stable in the Tg group (Figure 2D and E).

### Up-regulation of lysozyme secretion in transgenic goats

Lysozymes are small enzymes that attack the protective cell walls of bacteria. Lysozymes break carbohydrate chains, destroying the structural integrity of the cell wall. LZM expression in the Tg group was higher than in the WT group at 0.5 hours (*P<0.05*), after which it showed a steady upward trend (Figure 2F).

### Rapid filtration of inflammatory cells in transgenic goats

**Figure 3:**
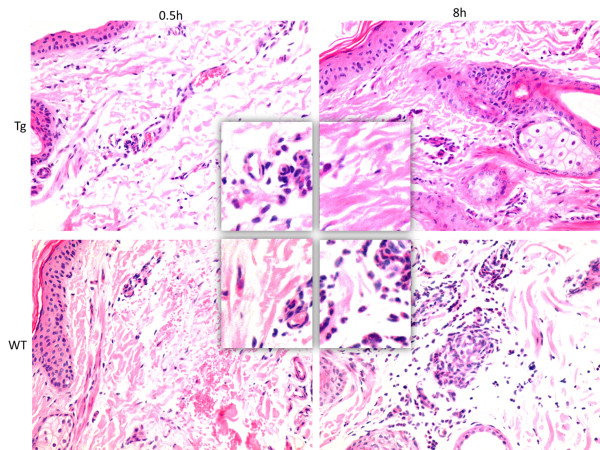
**Goats intradermally injected with 1 mg Pam3CSK4 in the ear.** Rapid filtration of inflammatory cells in transgenic goats. Pathologic changes were examined microscopically (×400). Tg = transgenic group, WT = wild-type group.

Goats were intradermally injected with Pam3CSK4 in the ear. Red blood cell infiltration was observed in both Tg and WT groups in the dermal connective tissue 0.5 hours after Pam3CSK4 challenge. Eight hours after Pam3CSK4 challenge, dermal damage was assessed and serious red cell and inflammatory cell infiltration was observed in the WT group. However, no pathological changes were observed in the Tg group 8 hours after treatment with Pam3CSK4 (Figure 3).

## Discussion

TLR2 is as a key player in the defence against organ damage and in innate immunity. It provides an important link between innate and adaptive immunity [10]. During bacterial infection, viral infection, host defence, allergic response, and transplant rejection, the Th1/Th2 cytokine balance is broken. The functional polymorphisms of TLR2 have been reported [11]. By binding with its ligand, TLR2 causes the activation of NF-κB, ultimately leading to the transcription of cytokines, chemokines, and co-stimulators. Once TLR2 is activated, TLR2-mediated Th2 responses are induced [12,13]. Studies have shown that the TLR2 pathway directly triggers the production of Th1 cytokines [14,15]. It has been reported that Th1 cytokines tend to induce pro-inflammatory responses and Th2-related cytokines, including IL-6 and IL-10, tend to produce anti-inflammatory responses and can counteract the Th1-mediated microbicidal actions [16]. In launching complex inflammatory reactions, IL-6 plays a crucial role as a pro-inflammatory cytokine. High levels of IL-6 can activate monocytes and macrophages [17]. IL-10 is the main anti-inflammatory factor [18].

During the onset of immune response, pro-inflammatory cytokines like IL-6 play a dominant role. During later phases, anti-inflammatory cytokine expression increases to prevent immune overreaction [19]. IFN-γ can inhibit Th2 cells and IL-10 can decrease levels of Th1-related cytokines, preventing the generation of monocyte-derived DCs [20]. Changes in IL-10, IL-6, and IFN-γ levels reflect the relationship between anti- and pro-inflammatory equilibrium and immune function [21]. IFN-γ and IL-10 expression was found to be up-regulated earlier in the Tg group than in the WT group. As a consequence, levels of pro-inflammatory cytokine decreased to their original values faster in the Tg group than in the WT group. Our results indicate that Pam3CSK4 stimulation might cause immunosuppression in the WT group. TLR2 transcription levels were found to be positively correlated with IL-10, IL-6, and IFN-γ levels in the Tg group. They were able to promote immune response. A slight up-regulation of IL-10 was observed at 0.5 hours post challenge. One reasonable explanation for this might be was negative self-regulation of the immune system. This regulation would reduce the intensity of cascade immune reactions, prevent monocyte- and macrophage-induced damage, and decrease the effects of the inflammatory responses that cause tissue damage. Recent studies have revealed that TLR2−/− mice experienced strong immune reactions [22]. Up-regulated expression of TLR2 in multiple organs was found to have a high mortality rate [23]. TLR2 was also found to induce neutrophil activation [24]. In the Tg group, Pam3CSK4-induced cytokine imbalances were resolved, and cytokine levels returned to original levels within 48 hours. This was earlier than in the WT group. Tissue sections showed that neutrophil activation enhanced rapid infiltration in Tg animals. Tissue damage attributable to severe inflammatory reaction was found to be inhibited by TLR2 over-expression.

NO is produced by a variety of cells. It has many functions involved in the response to microbe invasion and inflammatory injury. During macrophage activation, large amounts of NOS and super oxide anion radicals are released alongside NO and H_2_O_2_. The toxic effects of NO on nearby tissue and of NOS production on cells have been reported. Studies have found that the damage to cellular proteins, lipids, and nucleic acids caused by oxidation can contribute to immune reactions involving TLR2 [25]. One previous study indicated that activation of NF-κB was remarkably enhanced by exposed to NO in RAW264.7 cells [26]. The relatively low levels of NO were able to trigger downstream pathways and keep the defence mechanism functioning properly. In contrast, the activation of NF-κB was inhibited by high concentrations of NO [27]. The suppression of NF-κB activation decreased the inflammatory response and prevented tissue damage. Our results indicate that TLR2 over-expression was correlated to stable NO and MDA levels and moderate levels of oxidative stress. Recent reports have shown that lysozyme promotes the release of TLR2 stimulants from gram-positive organisms [28]. The results of the present study indicate that monocyte-macrophages were activated by Pam3CSK4 and that lysozyme secretion increased. Lysozyme content was higher in the Tg group than in the WT group. TLR2 over-expression was found to up-regulate lysozyme secretion and it may contribute to bacterial clearance.

## Conclusions

Goats over-expressing *Capra hircus* TLR2 were not found to differ significantly from wild-type goats in any physiological or biochemical respect. Among Tg animals, IL-6 levels were up-regulated and IFN-γ-induced immunity was depressed. NO concentrations remained relatively low. Enhancement of IL-10 expression occurred earlier than in wild-type. Inflammatory infiltrations and lysozyme secretion were increased. In vivo study indicated that the immune system responded quickly to protect tissue from damage. All of these results indicate that tissue damage can be prevented by over-expression of TLR2. This information may be useful to fostering disease resistance in goat breeding.

## Methods

### Animals

Superovulation, artificial insemination, intradermic injection, and blood collection were performed at the experimental station of the China Agricultural University. The study was carried out in strict accordance with the protocol approved by the Animal Welfare Committee of China Agricultural University (Permit Number: XK662).

### Production of *Capra hircus*in goats over-expressing TLR2

Total RNA was extracted from goat spleens using an RNA kit (OMEGABio-Tek, Doraville, GA, U.S.). cDNA was synthesized using M-MLV reverse transcriptase (Promega, Madison, WI, U.S.) in accordance with the manufacturer’s instructions and following the *Capra hircus* TLR2 gene sequence (GenBank: GU984768.1). The eukaryotic expression vector pN1 (Plasmid12193; Addgene, Cambridge, MA, U.S.) was used as a backbone for the transformation. It was generated by removal of the EGFP gene from pEGFP-N1. The construct sub-cloned from pIRES2-EGFP (Plasmid12193; Addgene, Cambridge, MA, U.S.) contained an IRES-EGFP fragment connected to pN1 after introduction of a LoxP sequence on either side. This expression vector is called p3S-LoxP. Both the *Capra hircus* TLR2 sequence and p3S-LoxP were digested before ligation. The new expression vector is here called p3S-LoxP-TLR2.

Healthy laso-shan dairy goats were put into synchronized estrus using CIDR (Pharmacia & UpjohnCompany, Rydalmere, Australia). Ova were collected from donors using superovulation. Zygotes were generated using in vitro fertilization. The zygotes were microinjected with linearized p3S-LoxP-TLR2 solution at concentrations of 5 ng/μL and 10 ng/μL in volumes of 5 pL. They were then transferred into the recipients’ oviducts. DNA was extracted from the ear tissue of each lamb at birth. To identify transgenic individuals, the following PCR primers were used: F: 5^′^- TCC AAA ATG TCG TAA CAA CTC CG - 3^′^; R: 5^′^ - AAA AAG AGA TGT TTC CCC AAG TGT T - 3^′^. The upstream primer was based on the CMV region and the downstream primer was based on foreign TLR2. For Southern blotting analysis, the PCR product of TLR2 was digested with Nhe I and Hind III (NEB, Beverly, MA, U.S.) and labelled with DIG (12647521;Roche Diagnostics, Mannheim, Germany) for use as a probe. Ear tissue section slides were paraffin-embedded and prepared for immunohistochemical analysis and TLR2 expression analysis. Anti-goat TLR2-FITC was used (ab59711; Abcam, Cambridge, U.K.).

### Testing of physiological and biochemical parameters

The blood biochemical parameters of transgenic and control goats were assessed at 120 days. Peripheral blood was collected and both blood cells serum biochemical parameters were assessed. Factors examined here included the white blood cell count (WBC), red blood cell count (RBC), and hemoglobin (HGB), hematocrit (HCT), serum total protein (TP), albumin (Alb), globulin (Glo), alanine aminotransferase (ALT), aspartate aminotransferase (AST), C-reactive protein (CRP), glucose (Glu), blood urea nitrogen (BUN), and triglyceride (TG) levels.

### In vitro Pam3CSK4-challenge

Mononuclear cells were isolated from the peripheral blood of 3 transgenic goats and 3 wild goats by density gradient centrifugation. Goat lymphocyte separation medium (TBD, Tianjin, China) was used. Cells were cultured in RPMI 1640 medium (Gibco, Grand Island, NY, U.S.) supplemented with 10% FBS (Gibco, Grand Island, NY, USA). For differential adhesion cultures, the medium was replaced once every 12 hours. During TLR2 receptor agonist experiments, monocytes and macrophages were cultured for 48 hours and stimulated at different times with 1 μg/mL Pam3CSK4 (InvivoGen, San Diego, CA, U.S.). Cell culture supernatants were collected, and the concentrations of IFN-γ, IL-10, IL-6, and LZM were measured using enzyme-linked immunosorbent assay (ELISA) kits. NO and MDA content were detected using a commercial kit (Jiancheng, Nanjing, China). All experimental operations were performed according to kit instructions. Total RNA was extracted from monocyte-macrophages. Real-time PCR was used to evaluate TLR2 expression. TLR2- and β-actin-specific primers were designed (TLR2 F: 5^′^ - TGC TGT GCC CTC TTC CTG TT - 3^′^, R: 5^′^- GGG ACG AAG TCT CGC TTA TGA A - 3^′^; β-actin F: 5^′^ - AGA TGT GGA TCA GCA AGC AG - 3^′^, R: 5^′^ - CCA ATC TCA TCT CGT TTT CTG - 3^′^). Relative expression was determined using the comparative 2^-△△CT^ method.

### In vivo Pam3CSK4-challenge

Live goats were intradermally injected with 1mg Pam3CSK4 in the ear. Ear tissues were collected at 0.5 hours and 8 hours after Pam3CSK4 stimulation. Samples were fixed with 4% paraformaldehyde in addition to the routine haematoxylin and eosin staining.

### Statistical analysis

Individual experiments were repeated three times. All data were subjected to analysis of variance using the GLM procedures of the statistical analysis system (SAS Institute, U.S.). All data are expressed as mean ± SEM. Differences were considered significant at *P< 0.05*.

## Competing interests

The authors declare that they have no competing interests.

## Authors’ contributions

SLD, KY, NL and ZXL initiated and conducted the experiments, coordinated the experimental design, analysis and interpretation of data and wrote the manuscript. SLD, BLZ, YCY, YFL, HJH, HTZ and MSC production of *Capra hircusin* goats over-expressing TLR2 and testing of physiological and biochemical parameters. SLD and KY conducted in vitro and vivo Pam3CSK4-challenge. All authors have read and approved the final manuscript.
